# Public Health

**Published:** 1887-06

**Authors:** 


### Reviews.

**Public Health.** The Lomb Prize Essays of the American Public Health Association. Second edition.

This book comprises four valuable papers which were presented at a late meeting of the association. The first is Prof. Victor C. Vaughan's admirable article, entitled "Healthy Homes and Food for the Working Classes." The second is "The Sanitary Conditions and Necessities of School Houses and School Life," by Dr. Lincoln. Dr. Sternberg treats of "The Disinfection and Individual Prophylaxis against Infectious Diseases." The last essay is by Dr. Ireland, on "Preventable Causes of Disease, Injury and Death in Manufactories and Workshops, &c." These essays are truly admirable. We wish they were in the hands of every doctor. In the introduction, it is stated that the association regrets its inability to furnish them free, but that the present book is published at cost. It can be had by addressing the secretary, Dr. J. A. Watson, Concord, N. H. Our readers can obtain them in pamphlet form at thirty cents; in book, cloth bound, for sixty-five cents; or more handsomely bound, at one dollar, including postage.

